# High Prevalence of *Echinostoma mekongi* Infection in Schoolchildren and Adults, Kandal Province, Cambodia

**DOI:** 10.3201/eid3003.240001

**Published:** 2024-03

**Authors:** Bong-Kwang Jung, Taehee Chang, Seungwan Ryoo, Sooji Hong, Jeonggyu Lee, Sung-Jong Hong, Woon-Mok Sohn, Virak Khieu, Rekol Huy, Jong-Yil Chai

**Affiliations:** MediCheck Research Institute, Korea Association of Health Promotion, Seoul, South Korea (B.-K. Jung, S. Ryoo, S. Hong, J. Lee);; Seoul National University Graduate School of Public Health, Seoul (T. Chang);; Seoul National University College of Medicine, Seoul (J.-Y. Chai);; Convergence Research Center for Insect Vectors, Inchon National University, Incheon, South Korea (S.-J. Hong);; Gyeongsang National University College of Medicine, Jinju, South Korea (W.-M. Sohn);; National Center for Parasitology, Entomology and Malaria Control, Ministry of Health, Phnom Penh, Cambodia (V. Khieu, R. Huy)

**Keywords:** *Echinostoma mekongi*, Cambodia, helminths, vector-borne infections, food safety, snails

## Abstract

A high prevalence of *Echinostoma mekongi* infection (13.9%; 260/1,876) was found among schoolchildren and adults in Kandal Province, Cambodia, by fecal examination, worm expulsion, and molecular analysis of *cox*1 and *nd*1 genes. The source of infection was consumption of *Pila* sp. snails, a finding confirmed morphologically and molecularly.

Echinostomiasis is a disease caused by infection with echinostome flukes (Echinostomatidae) and is characterized by intestinal inflammation accompanied by mucosal ulceration and bleeding (*1**,**2*). Echinostomiasis, a typical example of a foodborne helminthiasis, is contracted by consuming raw or improperly cooked snails, bivalves, fish, or amphibians (*1**,**2*). This disease has been neglected mainly because of underestimated prevalence and worm burden (global prevalence and burden unknown) as well as underrecognized clinical and public health significance. In South Korea and Japan, patients infected with the echinostome *Isthmiophora hortensis* reported gastrointestinal issues, and diagnosis was established after physicians extracted adult worms via gastrointestinal endoscopy (*1*).

*Echinostoma mekongi* was described as a new human-infecting echinostome that emerged in Kratie and Takeo Province, Cambodia, and identified through morphologic and molecular analyses (*3*). The adult flukes were recovered from persons residing along the Mekong River in these provinces, who reported abdominal discomfort, indigestion, and other gastrointestinal troubles (*3*). The metacercarial stage of *E. mekongi* was detected in freshwater snails, *Filopaludina martensi cambodjensis*, a popular food item in Pursat Province (*4*). We found a highly endemic area of *E. mekongi* infection in riverside villages of Kandal Province (surrounding Phnom Penh, the capital; population ≈1.27 million). Adult flukes were expelled after chemotherapy and purging and then analyzed morphologically and molecularly (*cox*1 and *nd*1 genes). Freshwater snails, *Pila* sp., were verified to be the source of infection, but the first intermediate host and the natural definitive host other than humans remain unknown.

## The Study

**Figure 1:**
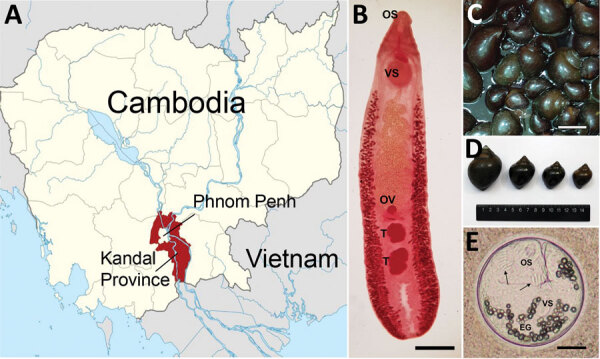
Study area and specimens of *Echinostoma mekongi* flukes and *Pila* sp. snails for study of *E. mekongi* infection in schoolchildren and adults, Kandal Province, Cambodia. A) Study area in Cambodia. B) Adult specimen of *E. mekongi* fluke expelled from a volunteer after chemotherapy and purging. Scale bar = 1.2 mm. C, D) *Pila* sp. snails purchased from a local market in Kandal Province, showing variable sizes. The presence of metacercariae in these snails was confirmed. Scale bar in panel D = 3 cm. E) Metacercaria of *E. mekongi* encysted in the tissue of a *Pila* sp. snail, showing its characteristic structures, including 37 collar spines (arrows), oral sucker, ventral sucker, and excretory granules. Scale bar = 50 m. EG, excretory granules; OS, oral sucker; OV, ovary; T, testis; VS, ventral sucker.

We collected fecal samples in May 2019 from 1,876 villagers, including 1,631 schoolchildren (794 boys and 837 girls, 5–19 years of age) and 245 adults (89 men and 156 women, 20–85 years of age), residing along the Mekong River in Kandal Province, Cambodia (Figure 1, panel A). We examined samples for helminth eggs by using the Kato-Katz thick-smear technique. The overall helminth egg-positive rate was 16.5%. The egg-positive rate of *E*. *mekongi* was 13.9% and markedly higher (>5 times) in schoolchildren (15.5%) than in adults (2.9%) (Table 1). *E. mekongi* eggs were operculated, oval to ovoid, yellowish, thin-shelled, and 102–130 (average 116) μm long and 62–90 (average 76) μm wide (n = 10). Other helminth species detected were *Opisthorchis viverrini* (0.9%), hookworms (0.7%), *Enterobius vermicularis* (0.7%), *Hymenolepis nana* (0.7%), *Trichuris trichiura* (0.3%), and others (Table 1).

**Table 1:** Results of fecal examinations in study of *Echinostoma mekongi* infection among schoolchildren and adults in riverside villages along the Mekong River in Kandal Province, Cambodia*

Age group	No. examined	No. (%) egg-positive cases									
		Any helminth egg	Em	Ov	Sm	Hw	Al	Tt	Ev	Hn	*Taenia* sp.
Schoolchildren	1,631	290 (17.8)	253 (15.5)	11 (0.7)	1 (0.1)	8 (0.5)	2 (0.1)	5 (0.3)	10 (0.6)	10 (0.6)	1 (0.1)
Adults	245	20 (8.2)	7 (2.9)	6 (2.4)	1 (0.4)	6 (2.4)	0	0	1 (0.4)	1 (0.4)	0
Total	1,876	310 (16.5)	260 (13.9)	17 (0.9)	2 (0.1)	14 (0.7)	2 (0.1)	5 (0.3)	11 (0.6)	11 (0.6)	1 (0.1)

*Em, *Echinostoma mekongi*; Ov, *Opisthorchis viverrini*; Sm, *Schistosoma mekongi*; Hw, hookworms; Al, *Ascaris lumbricoides*; Tt, *Trichuris trichiura*; Ev, *Enterobius vermicularis*; Hn, *Hymenolepis nana.*

We recruited 8 schoolchildren and 2 adult volunteers for the recovery of *E. mekongi* adult flukes (Table 2) and administered a single oral dose of 10–15 mg/kg praziquantel (Shin Poong Pharm. Co., https://shinpoong.co.kr/en/main/main.php), followed by purging with 20–30 g magnesium sulfate. We collected whole diarrheic stools 3 to 5 times and pooled them individually. We fixed adult flukes in 10% formalin, stained the samples with acetocarmine, cleared each in glycerin-alcohol, and mounted the samples in glycerin jelly. We kept some samples in 70%–80% ethanol for molecular analyses.

**Table 2:** Worm expulsion after praziquantel treatment and purging from volunteers positive for *Echinostoma mekongi* eggs in fecal examinations in study of *Echinostoma mekongi* infection in schoolchildren and adults, Kandal Province, Cambodia*

Age group and code no.	Age, y	No. *E. mekongi* eggs in Kato-Katz fecal smears†	No. adult *E. mekongi* fluke specimens expelled‡
Schoolchildren			
1	15	168	46
2	15	264	6
3	16	96	4
4	16	480	2
5	14	168	2
6	13	216	2
7	13	168	1§
8	12	48	1
Adults			
1	46	720	15
2	41	120	7§

*All case-patients were female. Fecal samples were collected individually 2–3 h after praziquantel administration and purging with MgSO_4_. †Eggs/g of feces; amount in a typical smear was assumed to be 41.7 mg. ‡All recovered worms were adults that contained eggs except for 38 of 46 worms from schoolchildren case 1, which were juvenile or young adults containing no or only a few uterine eggs. §Adult specimens of *Enterobius vermicularis* (120 female worms in schoolchildren no. 7 and 1 female worm in adult no. 2) were collected simultaneously

We recovered 48 adult and 38 juvenile specimens (86 in total) of *E. mekongi* flukes from the 10 volunteers (Table 2). Schoolchildren (n = 8) expelled a total of 64 worms (8 per child), and adults (n = 2) passed a total of 22 worms (11 per person) (Table 2). The adult flukes (Figure 1, panel B) were elongated and leaf-like, with small head collars and small collar spines (37 in 2 alternating rows; 5 corner spines), globular or slightly lobed testes, vitelline follicles not merging near the posterior end, and 7.7–11.2 (average 9.5) mm by 1.8–2.3 (average 2.1) mm in size (n = 10), all characteristic features of *E. mekongi* flukes (*3*).

We purchased *Pila* sp. snails (Figure 1, panels C and D) at a local market in Kandal Province and examined them for metacercariae by using the crushing method. We detected 10 metacercariae in 5 (7.1%) of 70 snails examined. The metacercariae (n = 5) were round, 165–188 (average 176) μm in diameter (Figure 1, panel E), and encysted with a thin, pinkish, refractile wall. The metacercariae were equipped with a total of 37 collar spines, oral and ventral suckers, excretory granules, and other internal organs.

We obtained mitochondrial cytochrome *c* oxidase 1 (*cox*1) and NADH dehydrogenase subunit 1 (*nd*1) gene sequences for molecular analyses of the adult flukes and metacercariae. We extracted the genomic DNA of each segment by using the DNeasy Blood and Tissue kit (QIAGEN, https://www.qiagen.com/us), following the manufacturer’s instructions. We performed PCR amplification and sequencing by using the primers (JB3 and JB13 for *cox*1 and JB11 and JB12 for *nd*1) and conditions described in a previous study (*5*). We constructed phylogenetic trees by using the maximum-likelihood method available in MEGA X (*6*) and also incorporating the Tamura-Nei model of nucleotide substitution with 1,000 bootstrap replications.

**Figure 2:**
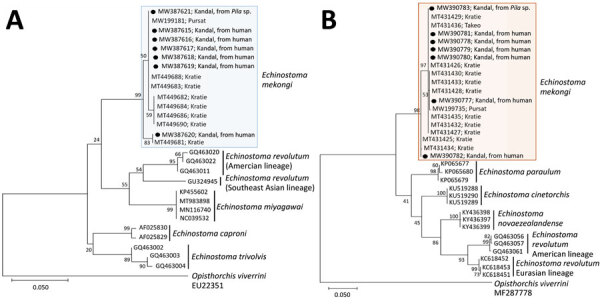
Phylogenetic trees of *cox*1 (A) and *nd*1 (B) genes of *Echinostoma mekongi* adults (n = 6) extracted from volunteers and metacercaria (n = 1) extracted from *Pila* sp. snails for study of *E. mekongi* infection in schoolchildren and adults, Kandal Province, Cambodia. Sequences from this study (shades boxes) are shown in comparison with other 37-collar-spined *Echinostoma* spp. (outgroup; *Opisthorchis viverrini*). The trees were constructed using the maximum-likelihood method, employing the Tamura-Nei model of nucleotide substitution with 1,000 bootstrap replications and viewed in MEGA X (https://www.megasoftware.net). GenBank accession numbers are given for all sequences. Scale bars indicate substitutions per site.

Partial sequences of *cox*1 (230 bp) (MW387615-MW387621) and *nd*1 (453 bp) (MW390777–83) genes in our samples (adult flukes and metacercariae) revealed strong identity with *E. mekongi* sequences (Figure 2, panels A and B). The phylogenetic tree of *cox*1 showed that our samples (n = 7) were tightly clustered (99.0%–100% identical) with *E. mekongi* (MT449688; human, Kratie Province, Cambodia) but separated from other 37-collar-spined echinostomes, including *E. caproni* (AF025830; 92.2%), *E. trivolvis* (GQ463003; 91.7%), *E. miyagawai* (KP455602; 90.2%–91.2%), and *E. revolutum* Southeast Asian (GU324945; 90.0%–91.0%) and American lineages (GQ463020; 89.8%). The phylogenetic tree of *nd*1 revealed also that our samples (n = 7) were closely aligned (98.7%–100%) with *E. mekongi* (MT431430; human, Kratie Province, Cambodia) but separated from other 37-collar-spined *Echinostoma* spp., including *E. paraulum* (KP065680; 88.7%–89.4%), *E. cinetorchis* (KU519289; 87.4%–88.1%), *E. novaezealandense* (KY436399; 86.9%–87.6%), and *E. revolutum* American (GQ463056; 86.3%–86.5%) and Eurasian lineages (KC618453; 86.2%–86.4%).

## Conclusions

Large trematode eggs, particularly, those of echinostomes, have been detected in various localities of Cambodia (*7**–**11*). In Pursat Province, echinostome eggs were found in 56 schoolchildren, and the worms expelled from 4 volunteers were assigned as *E. revolutum* by morphologic analysis (*7*). We think, however, that those worms might have been *E. mekongi* because *E. mekongi* and *E. revolutum* are morphologically close and almost indistinguishable (*3*). Molecular studies are necessary to draw a definite conclusion on the species of those echinostomes. In Oddar Meanchey Province, the eggs of echinostomes were detected in 13 persons, and the adult flukes expelled were confirmed to be *Echinostoma ilocanum* flukes, having 49–51 collar spines (*8*). Echinostome eggs were also detected in 71 persons in Kratie Province (*9*) and 52 persons in Takeo Province (*10*), and 6 volunteers were confirmed to be infected with *E. mekongi* flukes by morphologic and molecular analyses (*3*).

A previous study of persons in Kandal Province, Cambodia, found a high prevalence (46.5%; 106/228) of large trematode eggs (suggested to be *Echinostoma* spp.) among schoolchildren (5–18 years of age), but no adult worm recovery nor molecular analysis was performed (*11*). By the time of our study, it was confirmed that *E. mekongi* infection is highly prevalent among schoolchildren and adults in Kandal Province. The recovery of both juvenile and adult flukes may indicate the continuity of infection in this village. Freshwater snails of *Pila* sp. were proven to be the source of infection. It is speculated that *E. mekongi* infection might be prevalent not only in other localities of Cambodia but also in neighboring countries (Thailand, Laos, and Vietnam) along the Mekong River and its tributaries. Avoidance of consuming raw or undercooked *Pila* sp. snails is a preventive measure for this emerging parasitic infection in those areas. 
